# Complete genome sequence of two isolates from long-term serial cultivation of *Escherichia coli* JCM 5491

**DOI:** 10.1128/mra.00414-24

**Published:** 2024-08-28

**Authors:** So Umekage

**Affiliations:** 1 Department of Food and Nutrition, Hakodate Junior College, Hakodate, Hokkaido, Japan; 2 Department of Food and Nutrition, Koriyama Women's University, Koriyama, Fukushima, Japan; The University of Arizona, Tucson, Arizona, USA

**Keywords:** *Escherichia coli*, ATCC25922, JCM5491, long-term culture

## Abstract

Here, I report the complete genome sequences of two *Escherichia coli* JCM5491 mutants that were independently isolated from different lineages after approximately 9 years of long-term serial cultivation.

## ANNOUNCEMENT

*Escherichia coli* JCM 5491 is commonly used in food microbiological and antibacterial tests (1). Proper maintenance of bacterial strains is crucial for reliability. Short-read sequencing and long-read sequencing were performed to assess genomic mutations in *E. coli* JCM5491 after long-term serial cultivation.

*E. coli* JCM5491, obtained from the Japan Collection of Microorganisms, was inoculated onto two slant mediums made by plate count agar (1). These cultures were subcultured onto fresh slant mediums approximately every 2 weeks for at least 9 years at room temperature. A single colony from each culture was isolated by using the streak plate method (2) and preserved as glycerol stocks labeled LCT101 and LCT201. Genomic DNA for the short- and long-read sequencing was prepared independently and extracted from cultures grown in Luria–Bertani broth (3) using the NucleoSpin Microbial DNA kit (Macherey–Nagel GmbH & Co. KG). DNA fragments shorter than 25 kb were removed using a short-read eliminator kit (Nippon Genetics).

Long-read sequencing libraries for Oxford Nanopore sequencing were prepared using the SQK-RAD004 rapid sequencing kit. Sequencing was performed using the MinION Mk1b and an R9.4.1 flow cell (FLO-MIN106). Base calling was performed using the CPU version of guppy v.6.1.0 (Oxford Nanopore Technologies) with 16 CPU threads per caller. The quality check was performed using NanoPlot v.1.40.0 (4). The respective summarized statistics for LCT101 and LCT201 were as follows: number of reads, 618,883 and 33,763; read lengths of N_50_, 6,497 and 14,618; and total bases, 2,030,166,474 and 501,684,401. Reads longer than 13 kb with a quality greater than Q9 (equivalent to approximately 81 times and 58 times coverage, respectively) were assembled using Canu v.2.2 (5). The final circular single contigs (5,131,721 bp or LTC101 and 5,128,278 bp or LTC201; both 40 × coverage) were obtained by manually removing overlapping ends.

Short-read sequencing data were collected for hybrid error correction. Sequencing libraries were prepared using the MGIEasy FS DNA Library Prep Set (MGI), and 150-bp paired-end reads were sequenced on a DNBSEQ-G400RS (MGI) with a DNBSEQ-G400 high-throughput sequencing set FCL flow cell. The total number of reads was 6,044,763 for LTC101 and 7,363,929 for LTC201. Adapter trimming and quality filtering were performed using fastp v.0.19.6 (6). Hybrid error correction consisting of short-read mapping against the single contig using BWA-MEM v.0.7.17-r1188 (7) and polishing using Pilon v.1.24 (8) was repeated three times until no base corrections were reported. The completeness, contamination, and strain heterogeneity were assessed using CheckM (9). Genes were annotated using the DNA Data Bank of Japan (DDBJ) Fast Annotation and Submission Tool (10). An in-house Python script was used to rotate *dnaA* to the top for data submission to DDBJ. Default settings were used for tools, except where otherwise stated. Table 1 presents summary data for LTC101 and LTC201.

**TABLE 1:** Statistics of assembled whole-genome sequences of LTC101 and LTC201

	LTC101	LTC201
Total length (bp)	5,131,721	5,128,278
Coverage	40 ×	40 ×
Topology	Circular	Circular
Completeness (%)	99.46	99.46
Contamination (%)	0.53	0.53
Heterogeneity (%)	0	0
N_50_ (bp)	5,131,721	5,128,278
Gap ratio (%)	0	0
GC content (%)	50.5	50.5
No. of CDSs	4,713	4,702
Coding ratio (%)	86.9	86.9
No. of rRNAs	22	22
No. of tRNAs	89	89
No. of CRISPRs	0	0

## Data Availability

The complete genome sequences for LTC101 and LTC201 were deposited in the DDBJ under the accession numbers AP029248 and AP029249, respectively. The raw reads were deposited to the DDBJ Sequence Read Archive under the accession number PRJDB16273.
